# British Institutions for the Care of the Inebriate.—I

**Published:** 1903-09-26

**Authors:** 


### HOSPITAL ADMINISTRATION.

### CONSTRUCTION AND ECONOMICS.

### BRITISH INSTITUTIONS FOR THE CARE OF THE INEBRIATE.—I.

By Our Special Medical Commissioner.

#### The Scientific Study of Inebriety.

Inebriety stands foremost among morbid states hindering national progress and hampering individual development. It is a subject which has attracted the attention of all sorts and conditions of men, for either directly or indirectly the burden arising from the presence of the inebriate presses on all. The "Drink Question," as it is popularly termed, is many-sided, and can be approached by many different paths. Unfortunately ignorance, prejudice, and apathy have for long barred advancement into a clear understanding of the pathology of the condition of inebriety. Of recent years, however, the scientific method has been applied in the elucidation of the problem, and as a result of the serious investigation of the morbid characters of inebriety, rational measures for its prevention and arrest are being adopted.

Many notable works dealing with inebriety have appeared, and medical men generally are now devoting much attention to the subject.[1] The purely pathological aspects of the condition have also received considerable study;[2] and in the best scientific periodical literature much space is given to the results of research into the ætiological factors, clinical manifestations, and pathological basis of inebriety.[3]

The scientific investigation of inebriety can be viewed from many standpoints. It appeals to the morbid psychologist and alienist, it attracts the pure pathologist, and daily lies open to the observant clinician. It also raises considerations of the widest interest to the biologist and through the sociologist and moralist touches the most delicate researches into the problems of being. It is not our purpose here, however, to follow the results of the quest along these lines but rather to indicate their outcome in practical application to the prophylaxis and cure of inebriety, and particularly to such measures making for the restoration of the inebriate as are employed in the various institutions established for the treatment of this unfortunate class of case.

#### The Rôle of Legislation in the Treatment of Inebriety.

Britain has for many centuries occupied an unenviable position among the "drunken" nations of the earth, and from time to time spasmodic legislative effort has sought to arrest, mitigate, or regulate the evil. Until recent years the purely moral aspects of the condition dominated opinion, and the fundamental physiological and psychological bases of the pathological state were to a great extent neglected, and hence the application of rational measures for treatment has been long delayed. At last, however, with the progress of scientific knowledge directing power has gradually accumulated, and the energy of a well-based opinion has found expression in legislative measures.[4]

Dr. Branthwaite, in his excellent comparative study of legal enactments relating to inebriety in force in various countries, shows that they may be conveniently classified as follows:—

(1) *Penal.*—Measures regulating the punishment of occasional or habitual drunkenness by fine or by short terms of imprisonment.

(2) *Control* in penal establishments for lengthened periods.

(3) *Interdiction.*—Laws prohibiting the sale of liquor to persons who are known inebriates.

(4) *Guardianship.*—Acts regulating the appointment of some person or persons to act as guardian or guardians, who may be endowed with legal power over the person, and over the estate of an inebriate.

(5) *Control* in special institutions with a view to reformatory treatment:—(*a*) For the inebriate who makes voluntary application for admission. (*b*) By compulsory seclusion for the inebriate who refuses consent to treatment, and yet manages to keep out of the reach of the law. (*c*) For the inebriate who is a police court recidivist, or who has committed crime caused, or contributed to, by drink.

In this country, unfortunately, the application of remedial measures is much hampered by the limitation of our legal powers, and it must be admitted much of our machinery for effectually treating inebriety is inefficient, inadequate, and irrational. Public opinion, however, is rapidly awaking to the urgent need for increased legislative powers for dealing with the habitual inebriate, and it may be hoped sufficient and suitable institutions conducted on sound scientific lines, and under the best medical supervision will meet the needs of all classes.

#### British Statutes relating to the Care of the Inebriate.

The group of statutes based upon a scientific conception of the nature of inebriety, and known as the "Inebriates Acts," includes the Habitual Drunkards Act of 1879 and the Inebriates Acts of 1888, 1898, 1899, and 1900. Additional legislation dealing with the subject is also contained in the Prevention of Cruelty to Children Act of 1894, and in the new Licensing Act of 1902.[5]

A study of the statutes relating to inebriates and affording powers under which their care is now undertaken, shows

[1] Consult—"Alcoholism, a Study in Heredity," by Dr. G. Archdall Reid (1901). "Drink, Temperance, and Legislation," by Dr. Arthur Shadwell (1902). "The Temperance Problem and Social Reform," by Messrs. J. Rowntree and A. Sherwell. "Inebriety: Its Etiology, Pathology, Treatment, and Jurisprudence," by Dr. Norman Kerr. "Drunkenness," by Dr. George R. Wilson.

[2] See—"The Pathology of Alcoholism," by Professor Sims-Woodhead. *The British Medical Temperance Review.* Jan.–March, 1903. "The Influence of Stimulants and Narcotics on Health," by Sir Lauder Brunton, in "Disorders of Assimilation, Digestion, etc." 1901. "A Text-book of Pathology in Relation to Mental Diseases," by W. Ford Robertson, M.D. 1900.

[3] *The British Journal of Inebriety. The American Quarterly Journal of Inebriety.* Special Alcohol Number of *The Practitioner*, Nov. 1902.

[4] Consult the very valuable "Blue-Book," prepared by Dr. R. Welsh Branthwaite, H.M. Inspector under the Inebriates Act. "A Collection of British, Colonial, and Foreign Statutes relating to the Penal and Reformatory Treatment of Habitual Inebriates." 1902.

[5] "The Report of the Inspector under the Inebriates Acts, 1879 to 1900, for the year 1901" (London, 1902), contains a very useful outline of the laws which regulate the foundation and conduct of institutions for the inebriate, with an epitome of the powers generally relating to their treatment and control.

that they may be divided into two main divisions: (1) legislation applicable to persons who are inebriates without actually being law-breakers; and (2) legislative measures which can be put into force against persons who are inebriates and who commit offences punishable by fine or imprisonment.

It is very desirable therefore that no confusion should be allowed to arise in connection with the use of such terms as "inebriate homes," "retreats," and "reformatories." As Dr. Branthwaite has said: "There is no legal definition of 'inebriate home' in any Act; this may be considered as a generic term embracing all inebriate establishments of any type." It is very important, however, to differentiate between the "retreat" and the "reformatory." The former is designated purely for the reception of voluntary patients, persons who enter on their own application. The words "voluntary" and "retreat" must always be associated. The "reformatory," on the other hand, is established essentially for the compulsory detention of habitual drunkards, provided that they are also guilty of certain offences against law and order. Unless the distinction between these two types of institution be clearly understood, some persons will be deterred from application for admission into voluntary retreats on the assumption that they would be required to associate with police court and other committed cases. There is reason to believe that ignorance on this point has already caused some persons to hesitate in making application for admission.

For the greater part we shall have to deal with the ordinary licensed retreats and private homes; but we shall hope to indicate the need for the development of industrial retreats and colonies for inebriates; and we shall not be unmindful of the functions of the "Certified Inebriate Reformatory" and the "State Inebriate Reformatory."

### Institutional Control of the Inebriate.

In the treatment of inebriety suitable institutions have been found to be essential for the maintenance of such conditions as are necessary for success. In almost all civilised countries special establishments have of recent years sprung up where the control of the inebriate is undertaken and efforts made to secure restoration. In the present series of articles we are only concerned with British institutions for the care of the inebriate. These differ widely, not only in respect of the social class for which they cater, but also in the manner and method according to which they are supposed to be conducted. Only 18 licensed retreats exist in England and Wales. A very large number of "private" establishments which provide for inebriate cases are scattered through the country. For the sake of the inebriate and in the interests of the community it is most desirable that every such house, however large or however small, and irrespective of the number of patients received, should be open to State inspection. It is encouraging to find that philanthropic bodies are slowly undertaking the conduct of institutions for the inebriate classes. Ten years ago only two licensed retreats were conducted by societies whose object was philanthropy and not financial gain. To-day there are twelve such institutions, duly licensed and in active operation, or more than half of the total number of retreats. It may be of service to mention here the names of this honourable dozen. They are, in point of seniority, The Dalrymple Home, Rickmansworth, and The Grove, Fallowfield, Manchester; four connected with the Church of England Temperance Society—Ellison Lodge, at Herne Hill; Hancox, near Whatlington, Battle, Sussex; Congreave's Hall, at Cradley Park, Staffordshire; and Hamond Lodge, near King's Lynn—the famous Duxhurst at Reigate, in Surrey, superintended by Lady Henry Somerset; The Hermitage at South Cave, in East Yorkshire; the Brentry Retreat, directed by the Royal Victoria Homes Board; Victoria House, Thundersley, Essex, conducted by the Salvation Army; St. Veronica's Retreat at Chiswick, managed by a Roman Catholic sisterhood; and Spelthorne St. Mary, at Bedfont, Middlesex, under the care of the Sisters of St. Mary the Virgin, Wantage.

At the present time it is exceedingly difficult to obtain a complete list of institutions offering facilities for the effectual control of the inebriate.[6] The medical man desirous of finding a suitable institution for an inebriate patient is beset by considerable difficulties in endeavouring to select one from the advertisement columns of his medical periodicals. It is to meet this want that the present series of articles has been prepared. We are desirous, moreover, of securing improvement in the status of these institutions, and shall endeavour to procure such progress as may bring them into line with the best establishments dealing with other forms of mental disease.

It is clear, also, that much advantage would accrue if those connected with "retreats," or as we should prefer to term them "sanatoria for inebriates," could be afforded opportunities for comparison of methods and discussion as to variations in custom and procedure.

At the present time considerable difference in practice is unavoidable, and perhaps is not altogether to be regretted, but if progress is to be attained, it is essential that means should be found whereby information may be gathered, results compared, and new procedures made known.

In order to obtain trustworthy information for the busy practitioner, and stimulate a scientific study of the problems clustering round the perplexing subject of inebriety, the editor of The Hospital has arranged for the publication of a special series of articles, dealing with the more important British institutions devoted to the care of the inebriate. Each article will be based upon a special visit of inspection by an impartial expert, who will submit the results of a thorough investigation of each institution visited. It may be hoped that such careful and unprejudiced reports may be of much assistance to medical practitioners, and of service to the unfortunate class of inebriate patient, as well as prove of advantage to the various institutions dealt with.

*(To be continued.)*

[6] "Burdett's Hospitals and Charities, 1903," gives a list of 31. A small list is given in "The Medical Directory" (J. and A. Churchill); "The Medical Annual" (J. Wright and Co.) publishes a list; the novel little manual, "Where Shall I Send my Patient?" issued by "The Association of Medical Men receiving Resdient Patients" (E. J. Frampton, Bournemouth), gives references to 18, arranged according to locality; but the best hitherto available "Directory" has been issued in card form by "The Friends' Temperance Union" (Frank Dymond, 15 Devonshire Street, E.C.), and contains references to 41 institutions.

